# Supplementary material for: Silencing Nfix rescues muscular dystrophy by delaying muscle regeneration
Source: Nat Commun. 2017 Oct 20;8:1055. doi: 10.1038/s41467-017-01098-y (PMC5651883; doi:10.1038/s41467-017-01098-y)
Supplement: Supplementary file 1 — Supplementary Information [file 41467_2017_1098_MOESM1_ESM.pdf]

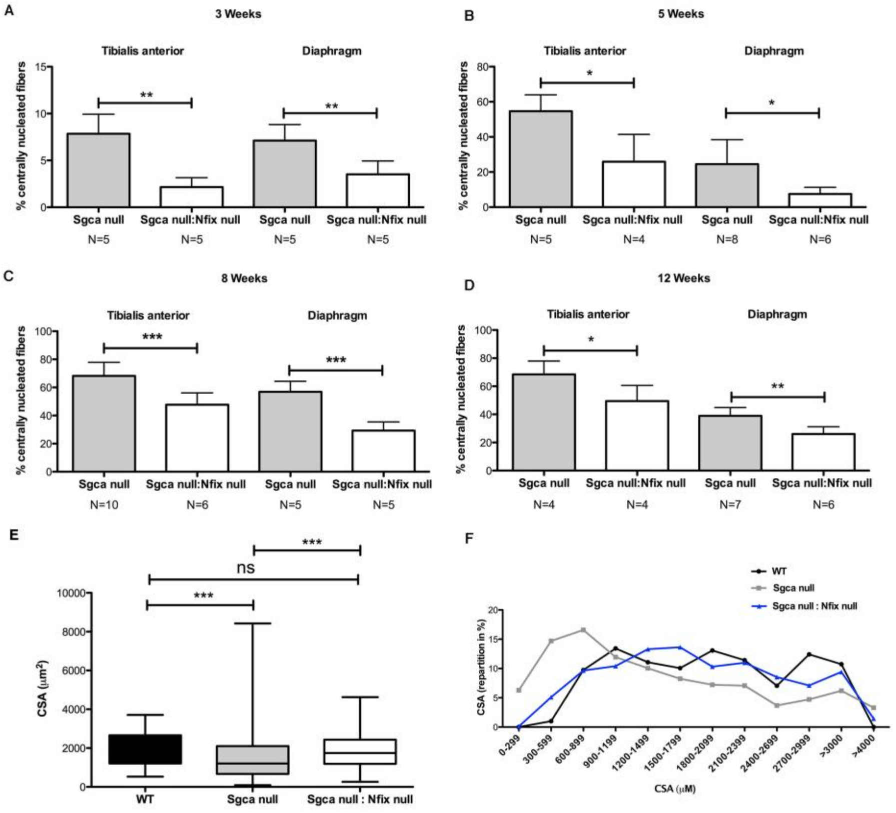

Supplementary Fig.1. The histophatological signs of the disease are improved in dystrophic mice lacking *Nfix*. Related to Fig. 1 and 2.

(A-D) Percentage of centrally nucleated myofibers in *Tibialis anterior* and Diaphragm muscles of *Sgca* null and *Sgca* null:*Nfix* null mice at 3 **(A)**, 5 **(B)**, 8 **(C)** and 12 **(D)** weeks; N are indicated in figure; mean± SD; *t*-Test, *P<0,05; **P<0,01; ***P<0,001;

(E, F) Myofiber cross sectional area distribution at 8 weeks; N=298 WT, 1273 *Sgca* null and 902 *Sgca* null:*Nfix* null fibers. **E**: Mean±whiskers from min to max; one way ANOVA with Bonferroni post test; ***P<0,001; ns: non significant.

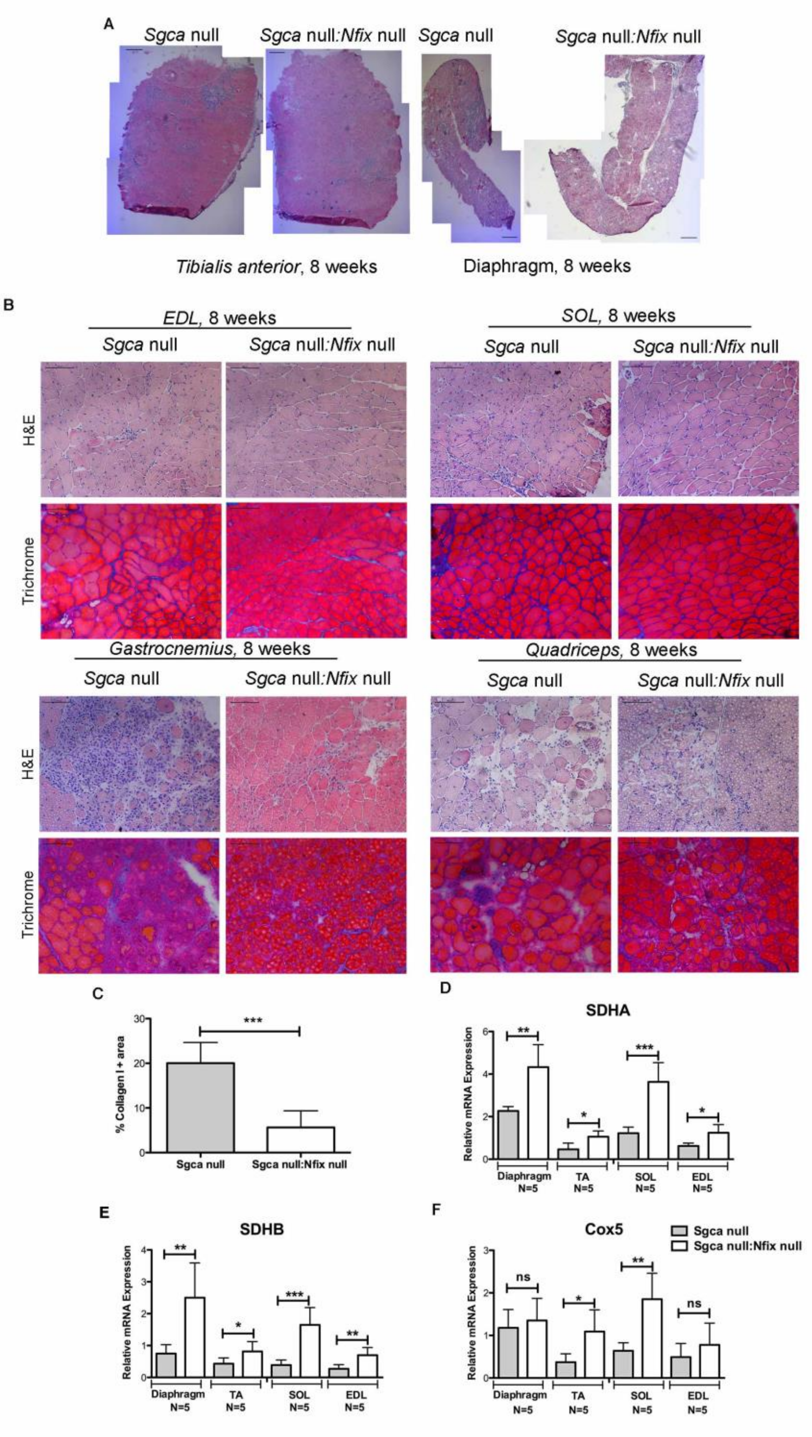

Supplementary Fig.2. Amelioration of different muscle types in dystrophic mice lacking *Nfix*; Related to Fig.2

(A) Entire H&E *TA* and Diaphragm muscle section reconstructions from *Sgca* null (N=23) and *Sgca* null:*Nfix* null (N=12) mice at 8 weeks;

(B) H&E and Trichrome stainings of *EDL*, *Soleus* (*SOL*), *Gastrocnemius* and *Quadriceps* muscles from *Sgca* null (N=3) and *Sgca* null:*Nfix* null (N=3) mice at 8 weeks. Scale bars 100μm;

(C) Quantification of Collagen I positive areas in *Tibialis anterior* muscle sections from *Sgca* null (N=5) and *Sgca* null:*Nfix* null (N=5) mice at 8 weeks; Mean±SD *t*-Test, ***P<0,001;

(D, E, F) Real Time PCR showing expression of SDH-A/B **(D, E)** and Cox5 **(F)** in *Sgca* null and *Sgca null:Nfix* null muscles at 3 weeks. N are indicated in figure. Mean±SD, *t*-Test, *P<0,05; **P<0,01; ***P<0,001; ns: non significant.

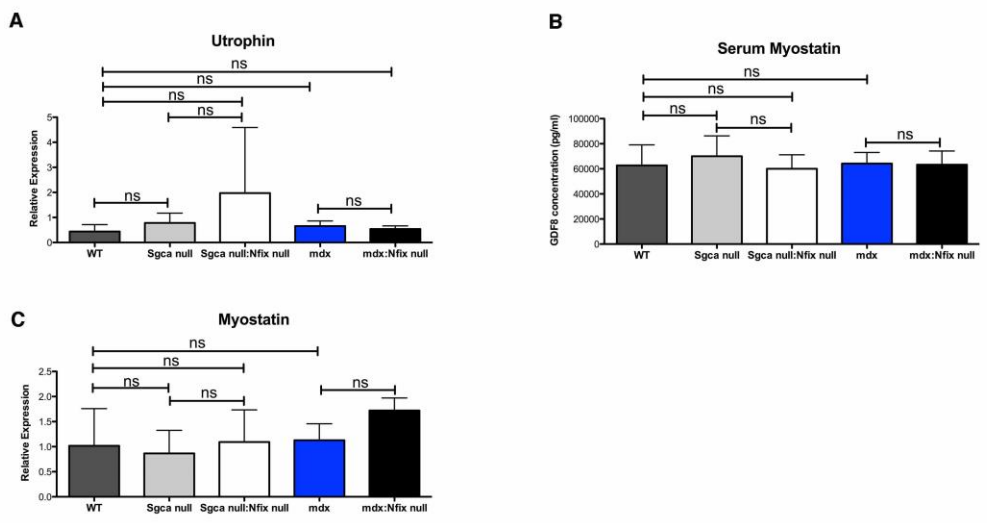

Supplementary Fig.3 Utrophin and Myostatin modulation is not at the basis of the phenotypic amelioration of the mdx:Nfix null and Sgca:Nfix null dystrophic mice; Related to Fig.2 and Fig.6.

(A) Real Time PCR analysis of *Utrophin* expression on N=4 WT, N=8 *Sgca* null, N=8 *Sgca* null:*Nfix* null, N=5 *MDX* and N=4 *MDX*:*Nfix* null gastrocnemius muscles. Mean±SD; one way ANOVA with Bonferroni post test; ns: non significant.

(B) Real Time PCR analysis of Myostatin expression on N=4 WT, N=8 *Sgca* null, N=8 *Sgca* null:*Nfix* null, N=5 *MDX* and N=4 *MDX*:*Nfix* null gastrocnemius muscles. Mean±SD; one way ANOVA with Bonferroni post test; ns: non significant.

(C) ELISA assay to detect Myostatin in serum on N=4 WT, N=7 *Sgca* null, N=7 *Sgca* null:*Nfix* null, N=5 *MDX* and N=4 *MDX*:*Nfix* null mice. Mean±SD; one way ANOVA with Bonferroni post test; ns: non significant.

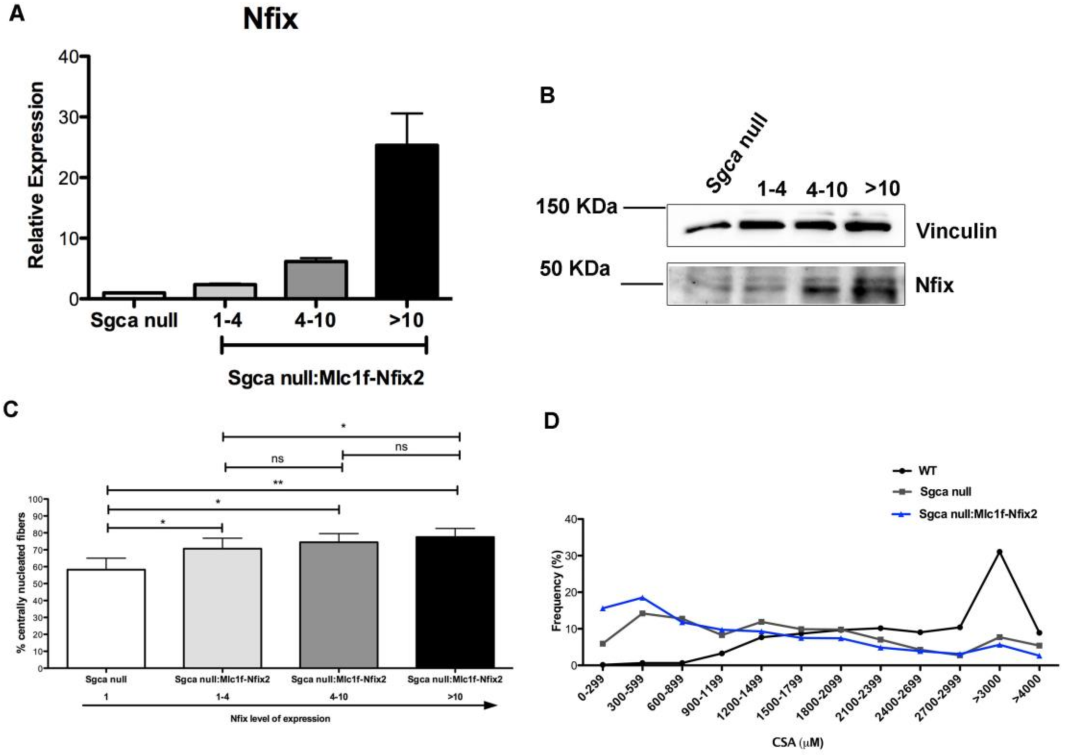

Supplementary Fig.4. The exacerbation of the dystrophic phenotype in the *Sgca* null:*Mlc1f-Nfix2* mice correlates with Nfix levels. Related to Fig.5.

(A) Real time PCR analysis of *Nfix* expression in *Sgca* null and *Sgca* null:*Mlc1f-Nfix2* mice subdivided in three different subclasses based on Nfix expression levels

(B) Western blot validation of Nfix overexpression in the three different subclasses of *Sgca* null:*Mlc1f-Nfix2* mice compared to *Sgca* null mice.

(C) Percentage of centrally nucleated myofibers in *Tibialis anterior* (TA) muscle sections from *Sgca* null and *Sgca* null:*Mlc1f-Nfix2* mice sub-classified based on Nfix overexpression levels at 8 weeks of age; N=5 *Sgca* null, 8 class (1-4), 3 class (4-10) and 7 class (>10) *Sgca* null:*Mlc1f-Nfix2* mice. Mean±SD, *t*-Test, *P<0,05; **P<0,01; ns: non significant.

(D) Myofiber cross sectional area distribution at 8 weeks of age; N=799 fibers for WT, 908 for *Sgca* null and 1026 for *Sgca* null:*Mlc1f-Nfix2* mice.

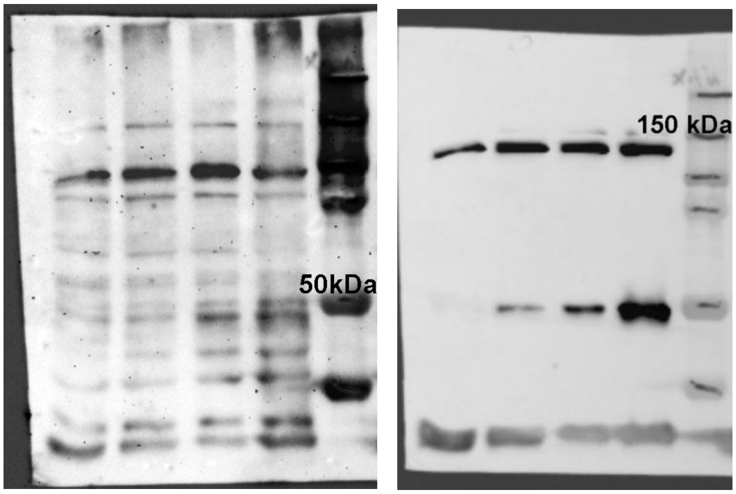

Supplementary Fig.5. Uncropped scans of Western Blots in Supplementary Fig.4

Uncropped Scans of Nfix (left panel) and Vinculin (right panel) western blots shown in Supplementary Fig.4
