# Supplementary material for: Silencing Nfix rescues muscular dystrophy by delaying muscle regeneration
Source: Nat Commun. 2017 Oct 20;8:1055. doi: 10.1038/s41467-017-01098-y (PMC5651883; doi:10.1038/s41467-017-01098-y)
Supplement: Supplementary file 3 — Description of Additional Supplementary Files [file 41467_2017_1098_MOESM3_ESM.pdf]

Description of Supplementary Files

File name: Supplementary Movie 1
Description: *Sgca* null:*Nfix* null mice perform better than *Sgca* null mice on treadmill test ; Related to Fig.3. 5X speed movie showing WT, *Sgca* null, Sgca null:*Nfix* null and *Nfix* null mice performance on treadmill test.
